# Discoid lateral meniscus: importance, diagnosis, and treatment

**DOI:** 10.1186/s40634-020-00294-y

**Published:** 2020-10-12

**Authors:** Jun-Ho Kim, Jin Hwan Ahn, Joo-Hwan Kim, Joon Ho Wang

**Affiliations:** 1 Department of Orthopedic Surgery, Seoul Medical Center, 156 Sinnae-ro, Jungnang-gu, Seoul, South Korea; 2 Department of Orthopedic Surgery, Saeum Hospital, Seoul, South Korea; 3 Department of Orthopedic Surgery, Samsung Medical Center, Sungkyunkwan University School of Medicine, 81 Irwon-ro, gangnam-gu, Seoul, 135-710 South Korea; 4 Department of Health Sciences and Technology and Department of Medical Device Management and Research, SAIHST, Sungkyunkwan University, Seoul, South Korea

## Abstract

Discoid lateral meniscus (DLM) is a common anatomic variant in the knee typically presented in young populations, with a greater incidence in the Asian population than in other populations. As DLM is a congenital anomaly, the ultrastructural features and morphology differ from those of the normal meniscus, potentially leading to meniscal tears. Snapping and pain are common symptoms, with occasional limitations of extension, in patients with DLM. Examination of the contralateral knee is necessary as DLM affects both knees. While simple radiographs may provide indirect signs of a DLM, magnetic resonance imaging (MRI) is essential for diagnosis and treatment planning. Although DLM was traditionally classified into three categories, namely, complete, incomplete, and Wrisberg DLM, a recent MRI classification provides useful information for surgical planning because the MRI classification was based on the peripheral detachment in patients with DLM, as follows: no shift, anterocentral shift, posterocentral shift, and central shift. Asymptomatic patients require close follow-up without surgical treatment, while patients with symptoms often require surgery. Total or subtotal meniscectomy, which has been traditionally performed, leads to an increased risk of degenerative arthritis; thus, partial meniscectomy is currently considered the treatment of choice for DLM. In addition to partial meniscectomy, meniscal repair of peripheral detachment is recommended for stabilization in patients with DLM to preserve the function of the meniscus. Previous studies have reported that partial meniscectomy with or without meniscal repair is effective and shows superior clinical and radiological outcomes to those of total or subtotal meniscectomy during the short- to long-term follow-up. Our preferred principle for DLM treatment is reduction, followed by reshaping with reference to the midbody of the medial meniscus and repair as firm as possible.

## Introduction

Discoid meniscus is a congenital variant of the knee joint that involves morphological and structural deformation, with potential meniscal instability. Discoid meniscus was first reported in 1889 by Young [73] following cadaver dissection. Discoid lateral meniscus (DLM) is commonly observed, with an approximate incidence rate ranging from 0.4% to 17%, while discoid medial meniscus is rarely detected, with an incidence of 0.06% to 0.3% [20, 25, 29, 30, 35, 70]. A higher prevalence has been reported in the Asian populations (10–15%) than in the Western populations (3–5%) [23, 32, 35, 38, 42]. Bilateral involvement is observed in 15–25% of the patients with DLM [22, 34, 53, 57, 70]. However, Ahn et al. [3] investigated the magnetic resonance imaging (MRI) review of the contralateral knees in patients with unilateral symptomatic DLM who underwent arthroscopic surgery and reported that 97% of the patients had a complete or incomplete DLM, although the study population was restricted to young Asian male patients. Thus, it is difficult to confirm the true incidence of DLM owing to the large number of asymptomatic cases and the limitations of diagnostic accuracy.

The DLM is vulnerable to tearing because of its morphological and structural characteristics, leading to the manifestation of symptoms, such as pain, snapping, or limited extension, which commonly develop in response to peripheral detachment [7, 20, 22]. The current preferred treatment for symptomatic patients with DLM is meniscal reshaping with or without meniscal repair rather than total or subtotal meniscectomy because the latter leads to deterioration of the lateral compartment [1, 4, 10, 55]. A thorough understanding of DLM is required for successful treatment, and preoperative planning using MRI is crucial for surgeons [5]. Thus, this review summarizes the current knowledge on the anatomy, classifications, clinical features, imaging studies, and treatment of DLM; we also comment on our preferred treatment strategy.

### Anatomy

The normal meniscus forms at the 8th week and attains mature anatomic morphology at the 14th week of fetal development [12]. Smillie [62] postulated that a discoid shape may develop owing to failure of absorption of the inner part of the menisci in the developing embryo. However, his theory was rebutted because the discoid morphology has not been detected in human or animal embryos, and only normal development of the menisci was observed [17, 33]. The discoid meniscus may be a congenital anomaly; this theory is supported by reports of the prevalence of the discoid meniscus in twins and by studies reporting on familial inheritance of the variant [19, 24]. The entire meniscus has vascular supply at birth, while the central third of the meniscus becomes avascular by 10 years of age as the vascular supply recedes during maturation [17].

The normal lateral meniscus is circular in shape, with an average thickness of 4.5 mm and width of 11 mm, and covers nearly 70% of the lateral tibial plateau [32]. The lateral meniscus shows greater excursion than the medial meniscus with respect to the range of motion because it is discontinuously attached to the joint capsule [25]. At the posterolateral attachment of the lateral meniscus, the popliteomeniscal fascicles, specifically the posterosuperior and anteroinferior fascicles, connect the posterior horn of the lateral meniscus to the popliteal tendon, forming the popliteal hiatus instead of the meniscocapsular tissue. The posterior part of the lateral meniscus is attached relatively firmly; these attachments are augmented by anterior (Humphrey) and posterior (Wrisberg) meniscofemoral ligaments, running anterior and posterior to the posterior cruciate ligament, respectively.

Morphologically, the DLM has increased thickness, enlarged surface, and decreased peripheral vascularity compared to the normal lateral meniscus [17, 50]. With respect to the ultrastructure of the DLM, a heterogenous and disorganized circumferential network of collagen a decreased number of collagen fibers are observed [14, 18, 51]. Owing to the morphological and ultrastructural abnormalities, the DLM is prone to tearing [17]. Recent case reports have described regeneration after meniscal reshaping of the DLM, which suggests that the DLM may be deformed to compensate for instability or structural abnormalities [15, 28, 64, 66].

### Classification

Watanabe et al. [68] first proposed the classification for the DLM in 1969 based on the arthroscopic appearance. They classified the DLM as complete, incomplete, and Wrisberg DLM, according to the degree of coverage of the lateral tibial plateau and presence of normal posterior attachment. Complete DLM (type I) refers to a block-shaped meniscus covering the entire tibial plateau with normal posterior attachment. Incomplete DLM (type II) refers to a semilunar-shaped meniscus covering up to 80% of the tibial plateau with normal posterior attachment. Wrisberg type DLM (type III) is more normally shaped but is unstable compared to the normal meniscus because it lacks the usual posterior attachments (coronary ligament or popliteomeniscal fascicles) and has the posterior meniscofemoral ligament (Wrisberg ligament) only. It is postulated that hypermobility at the posterior horn in the Wrisberg type DLM may induce the “snapping knee syndrome” when the knee is in the extended position [43, 49]. However, several studies [3–5, 7, 47, 71] have reported that the Wribserg type DLM was not identified in their case cohorts, and Ahn et al. [5] proposed that the Wrisberg type DLM may develop owing to instability because of peripheral detachment. The controversy regarding the actual presence or etiology (traumatic or inherent) of Wrisberg type DLM still remains. Although the Watanabe classification is the most commonly used classification, the actual usefulness of this classification system for surgical decision-making or planning remains somewhat questionable.

In 2004, Klingele et al. [40] described a new classification that focused on peripheral rim stability, and the DLM was classified according to the morphology, peripheral rim stability, and presence or absence of meniscal tears based on the arthroscopic findings.

**Fig. 1:**
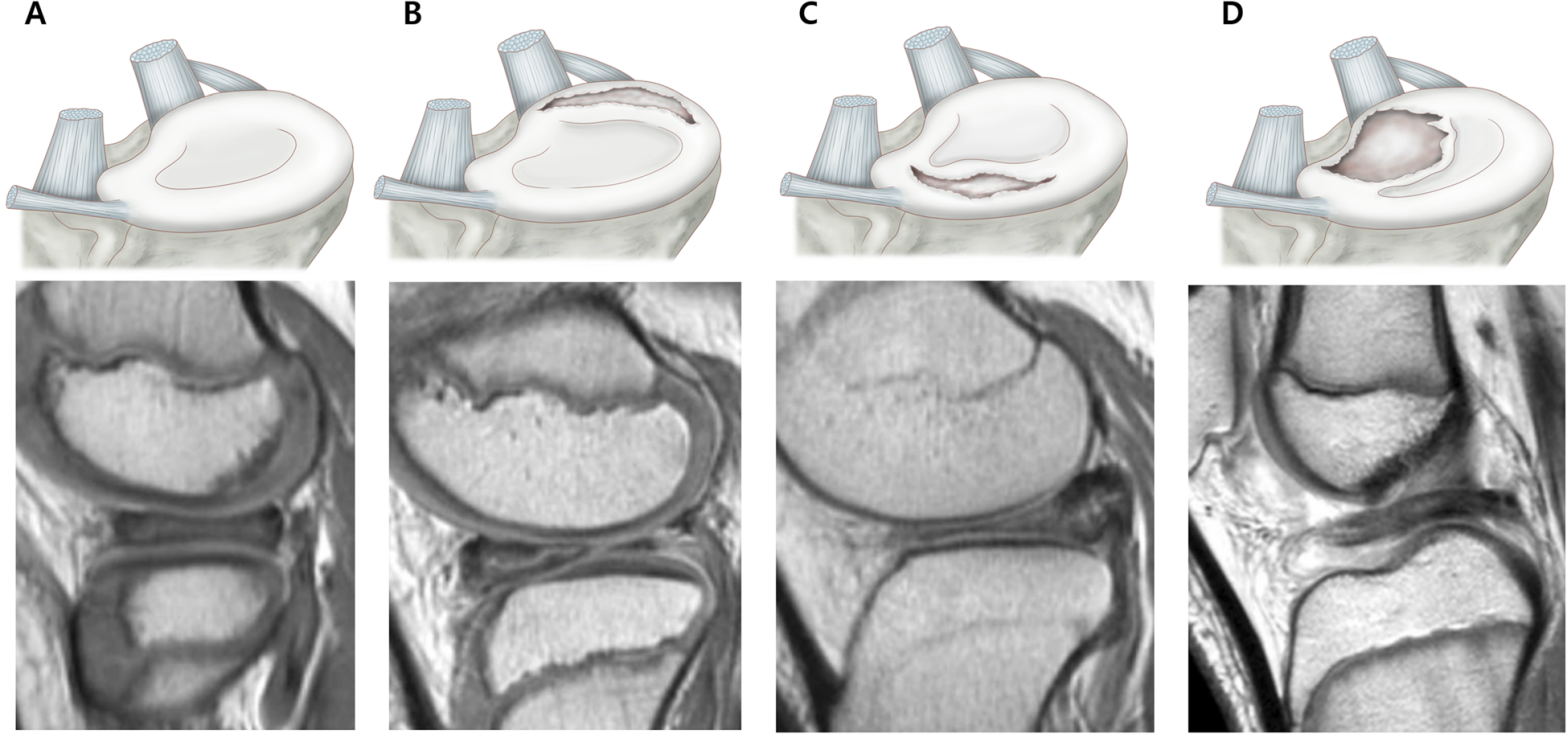
Magnetic resonance imaging classification of the discoid lateral meniscus (DLM) based on peripheral detachment. **a** No shift, **b** anterocentral shifting corresponding to a longitudinal tear in the posterior horn, **c** posterocentral shifting corresponding to a longitudinal tear in the anterior horn, and **d** central shifting corresponding to posterolateral corner loss

Ahn et al. [5] proposed an MRI-based classification in which the DLM was classified into four categories (no shift, anterocentral shift, posterocentral shift, and central shift) based on the concept of “meniscal shift” from peripheral detachment of the DLM. They reported that meniscal shift was associated with peripheral longitudinal tears that developed anteriorly or posteriorly, potentially resulting in a torn meniscal fragment that shifted freely. They also correlated the four categories in MRI scans with tear sites in the arthroscopic findings and demonstrated that anterocentral, posterocentral, and central DLM shifts were correlated with the longitudinal tears in the meniscocapsular junction of the anterior horn and posterior horn and posterolateral corner loss, respectively (Fig. 1). The MRI classification provides complementary and useful information to surgeons for appropriate surgical planning and decision-making.

### Clinical features

A stable DLM is incidentally detected in patients with asymptomatic or subtle clinical presentations, such as clicking [34]. As patients with a stable DLM may become symptomatic on insidious onset because the DLM is susceptible to tearing, careful serial examination is necessary [34]. An unstable DLM is associated with classical symptoms of snapping or popping with pain, effusion, giving way, or locking. On physical examination, the patient may present effusion, a lack of terminal extension, anterolateral bulging at full flexion, a positive McMurray test, or joint line tenderness. Children younger than around 10 years of age tend to present with spontaneous intermittent snapping and inability to achieve full extension, whereas adults may present with pain and mechanical symptoms [20, 42]. In patients with suspected DLM, examination and serial follow-up of the contralateral knee are important owing to the higher bilateral incidence [53]. A recent study found that patients with a DLM tear have a risk of development of a similar condition in the contralateral knee [31]. The overall sensitivity of clinical examination for DLM diagnosis varies from 29% to 93%, depending on the examiner’s experience and knowledge; thus, further imaging studies are necessary [35, 41, 65].

### Imaging studies

#### Plain radiography

Patients with DLM often show normal plain radiography findings but may also show subtle indirect signs; thus, radiology is considered a supplementary modality for the diagnosis of DLM. The indirect signs of DLM include widening of the lateral joint space, squaring of the lateral femoral condyle, cupping of the lateral tibial plateau, lateral tibial eminence hypoplasia, elevation of the fibular head, and condylar cutoff sign [26, 27, 43, 52, 57]. Recent studies showed that the condylar cut-off sign on tunnel-view radiography has a high specificity, indicating its diagnostic utility for complete DLM in children and adolescents [26, 27, 52]. In addition, Kim et al. [37] reported a significant association between meniscal tear of the contralateral knee and the presence of one or more than two indirect signs on plain radiographs.

#### MRI

MRI is an important diagnostic tool adjunct to the clinical features of the DLM and a useful modality for diagnosing DLM and for the assessment of the peripheral detachment in the DLM. Samato et al. [60] proposed criteria including a ratio of the minimal meniscal width to maximal tibial width on the coronal plane of more than 20% and a ratio of the sum of the width of both lateral horns to the meniscal diameter on the sagittal plane of more than 75% for the accurate diagnostic of a DLM on MRI. A minimal width of > 15 mm on the coronal plane and three or more 5-mm thick consecutive sagittal slices showing continuity between the anterior and posterior horns of the lateral meniscus are also suggestive diagnostic criteria for the presence of a DLM [13, 61].

MRI can also provide information on meniscal substance, meniscal tear, and the presence of accompanying osteochondritis dissecans. MRI findings such as the signal change of intra-meniscal substance and parameniscal edema have been described as an indirect sign of meniscal tear in patients with symptomatic DLM [58]. Meniscal deformation, such as abnormal infolding or buckling and meniscal shifting, are valuable indicators for the peripheral detachment of the DLM [58]. In this respect, Ahn et al. [5] proposed the MRI classification of the DLM for providing information for surgical planning with respect to the direction of meniscal shifting based on peripheral detachment of the DLM in cases of meniscus repair. Horizontal tears are commonly observed in the DLM not only in older patients, but also in children because of the characteristic fragile structure of the DLM; the presence of horizontal tears is concerning as the treats may extend to the periphery [58]. However, it is difficult to confirming the instability of the DLM on MRI because of the dynamic features of DLM with peripheral detachment and incomplete DLM mimicking a normal meniscus. Thus, MRI should not be considered markedly superior for the diagnosis of DLM compared to clinical examination, particularly in light of the significant differences in the sensitivity for detecting the DLM between the two methods (MRI, 39.8%; clinical examination, 88.9%) [41]. These modalities should be used concurrently to aid decision-making regarding the management of patients with DLM.

#### Arthroscopy

Arthroscopy may be necessary to confirm the diagnosis of DLM in symptomatic patients with peripheral detachment or instability, although tears and DLM are not visible in MRI scans.

### Treatment

#### Decision making

**Fig. 2:**
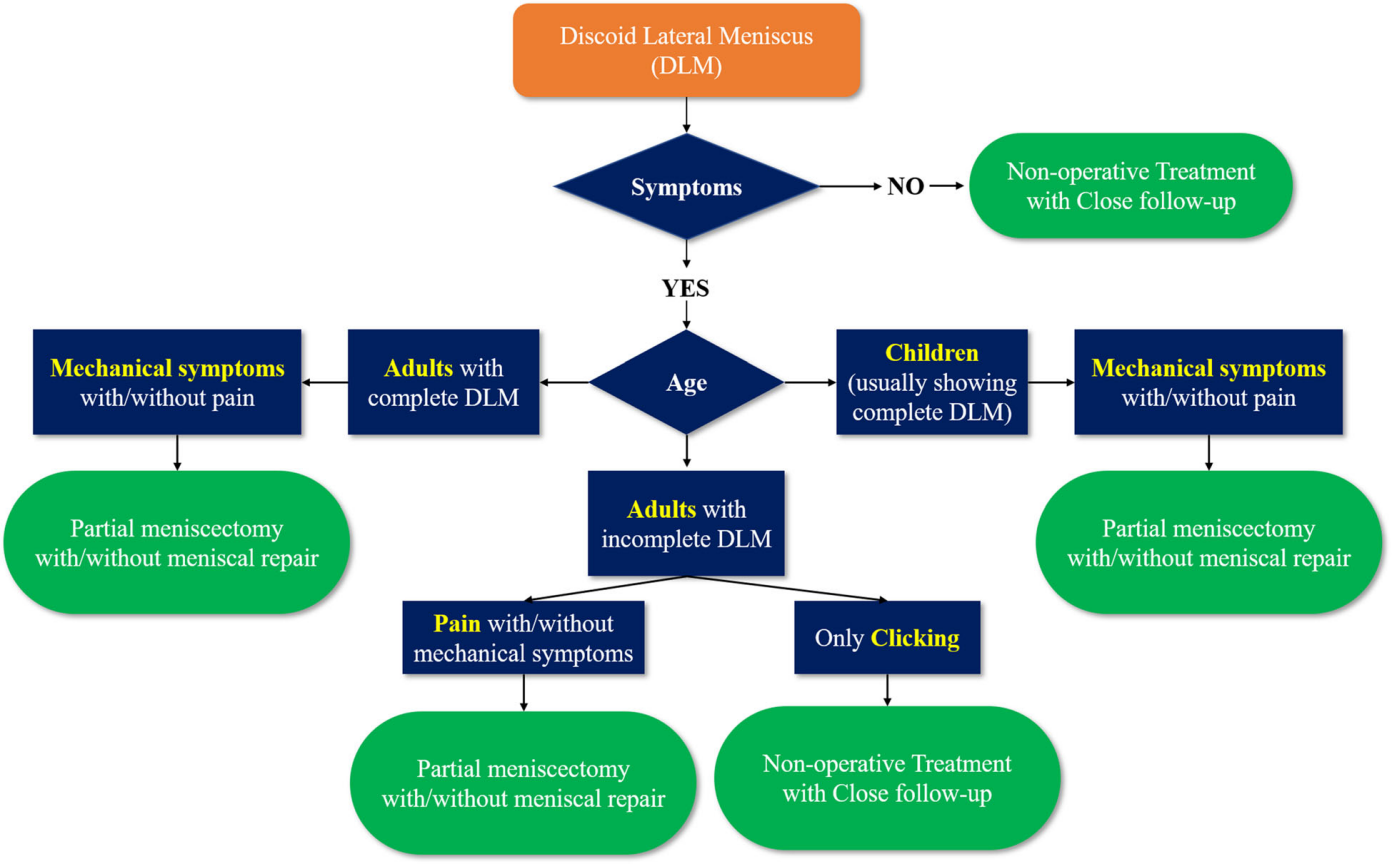
Treatment algorithm for patients with discoid lateral meniscus at our author’s institution

The principle of treatment for patients with DLM is simple; asymptomatic patients with incidentally detected DLM require non-operative treatment with periodic follow-up, while symptomatic patients with DLM may need operative treatment, such as arthroscopic partial meniscectomy with or without meniscal repair, and subtotal or total meniscectomy. Subtle symptoms, such as snapping remain under debate whether to fix immediately or to wait for significant symptoms to develop [35, 44]. Previous review articles on the DLM indicated that snapping knee with no other symptoms could wait until the condition becomes significantly symptomatic because the knee may have adapted to the DLM anatomy [35, 42, 44, 70]. However, we consider that the surgical indication should depend on the age of the patients with DLM. Children with mechanical symptoms, such as snapping and clicking often present with a complete DLM, which is prone to tearing. Thus, careful and thorough evaluation is necessary because a DLM with peripheral tears is often detected during arthroscopy in pediatric patients with complete DLM presenting with only snapping. Meanwhile, older patients presenting slight clicking or snapping without pain or locking symptoms usually have an incomplete DLM; thus, close follow-up is required rather than operation because they have adapted to the knee with a DLM. However, if any patients experience pain or locking symptoms, surgical intervention is warranted (Fig. 2).

#### Surgical treatment

Total meniscectomy has been historically considered the treatment of choice for symptomatic DLM in order to avoid the risk of inherent anomaly in the remnant meniscal tissue [11]. However, studies have reported that resecting the meniscus results in a high risk of lateral compartment osteoarthritis and poor clinical outcomes [21, 55]. Considering the nature of meniscal function, the aim of treatment planning should be to preserve the meniscus tissue as well as possible.

**Fig. 3:**
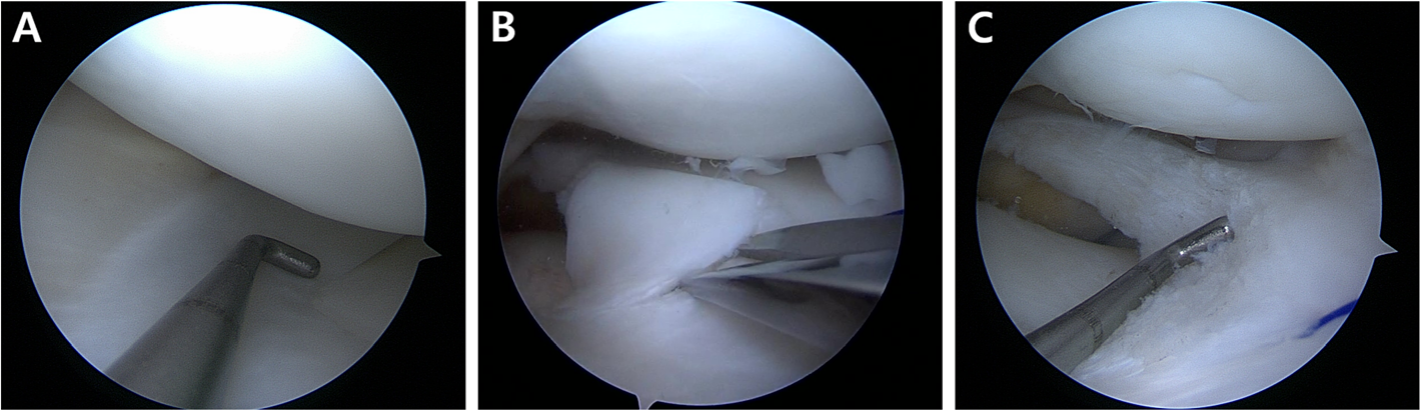
When performing partial meniscectomy for meniscal reshaping for patients with discoid lateral meniscus (DLM), the midbody of the medial meniscus is considered the reference for the remaining peripheral rim of the DLM (**a**). In addition to the arthroscopic instruments, the iris scissors are useful for resecting and trimming the anterior and middle portions of the DLM (**b**, **c**)

Partial meniscectomy, referred to as meniscal reshaping, meniscoplasty, or saucerization, is currently favored as the treatment of choice for patients with symptomatic stable DLM [4, 54, 70]. This technique aims to ensure that the DLM is of a normal size and shape so as to provide adequate meniscus function without re-tearing. The width of the remaining peripheral rim of the DLM is an important issue during partial meniscectomy; studies have recommended various guidelines, suggesting 4–5 mm and 6–8 mm as the adequate width [1, 4, 35, 47, 67, 70]. There are concerns that impingement or re-tears may develop in the remnant meniscal tissue [1, 35, 70]. Conversely, Yamasaki et al. [69] found that a remaining peripheral rim measuring less than 5 mm in width led to degenerative changes. Furthermore, Kinugasa et al. [39] reported that DLM repair without partial meniscectomy showed good clinical outcomes without re-tear at 2-year follow-up. Thus, there remains no clear consensus regarding the extent of peripheral rim preservation and what anatomic reference should be considered during arthroscopic partial meniscectomy. Recently, Kim et al. [37] suggested that the midbody of the medial meniscus should be considered the reference point for the remaining peripheral rim in partial meniscectomy for patients with complete DLM; this is identical to our practice regarding the reference (Fig. 3). Partial meniscectomy can be performed via the one-piece or piecemeal techniques [4]. In addition to the arthroscopic instruments, the iris scissors are useful for resecting and trimming the anterior and middle portions of the DLM (Fig. 3) [4].

**Fig. 4:**
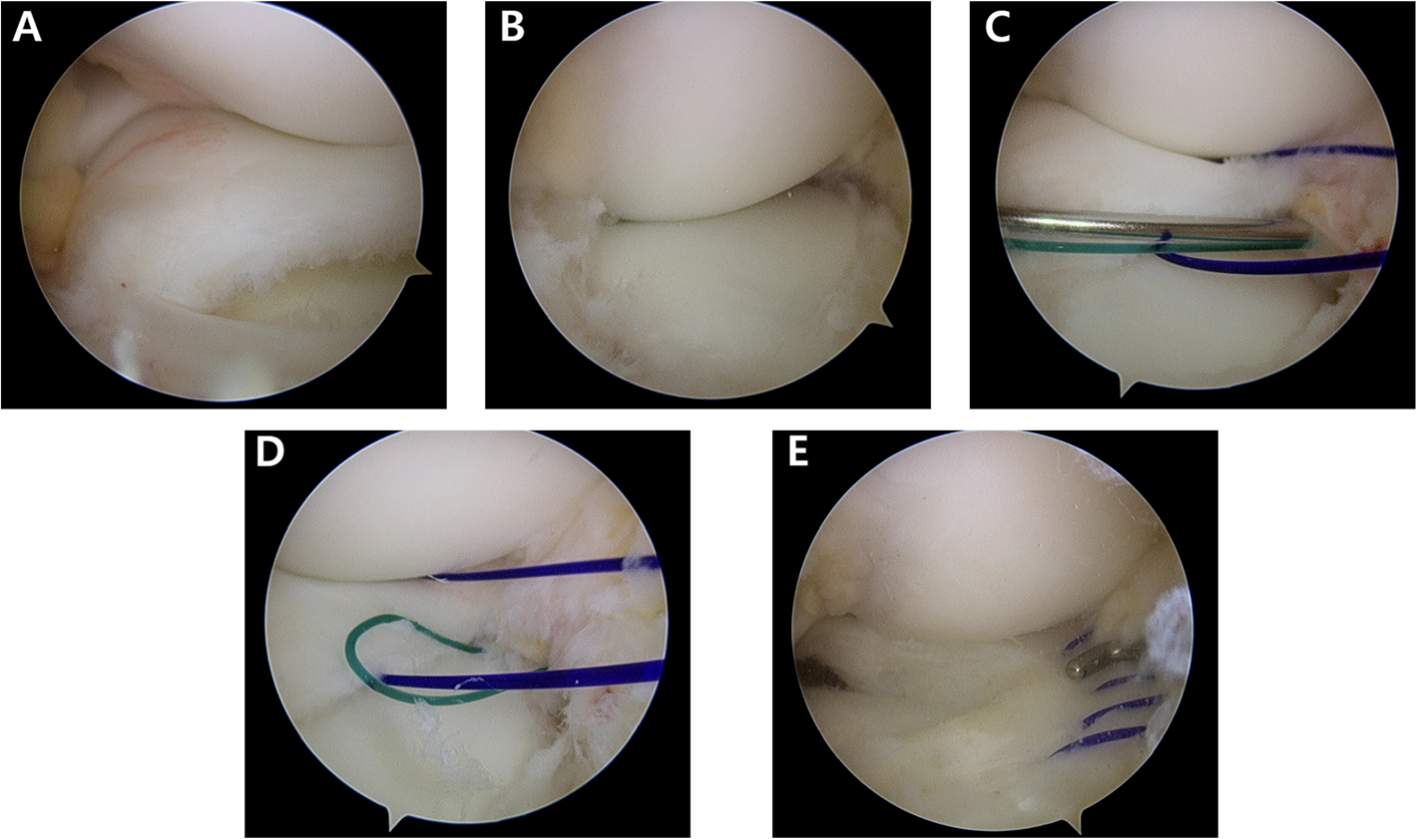
Arthroscopic view recorded during the treatment of the posterocentral shift type of complete discoid lateral meniscus (DLM) on the left knee of a 10-year old girl. The torn complete DLM was shifting to posterocentrally during knee flexion owing to a longitudinal tear of the anterior horn (**a**, **b** First, reduction was performed wherein sutures were placed using the modified outside-in technique (**c**, **d**); partial meniscectomy of DLM was then performed with reference to the midbody of the medial meniscus, followed by meniscal repair using a modified outside-in technique with five stitches (**e**)

**Fig. 5:**
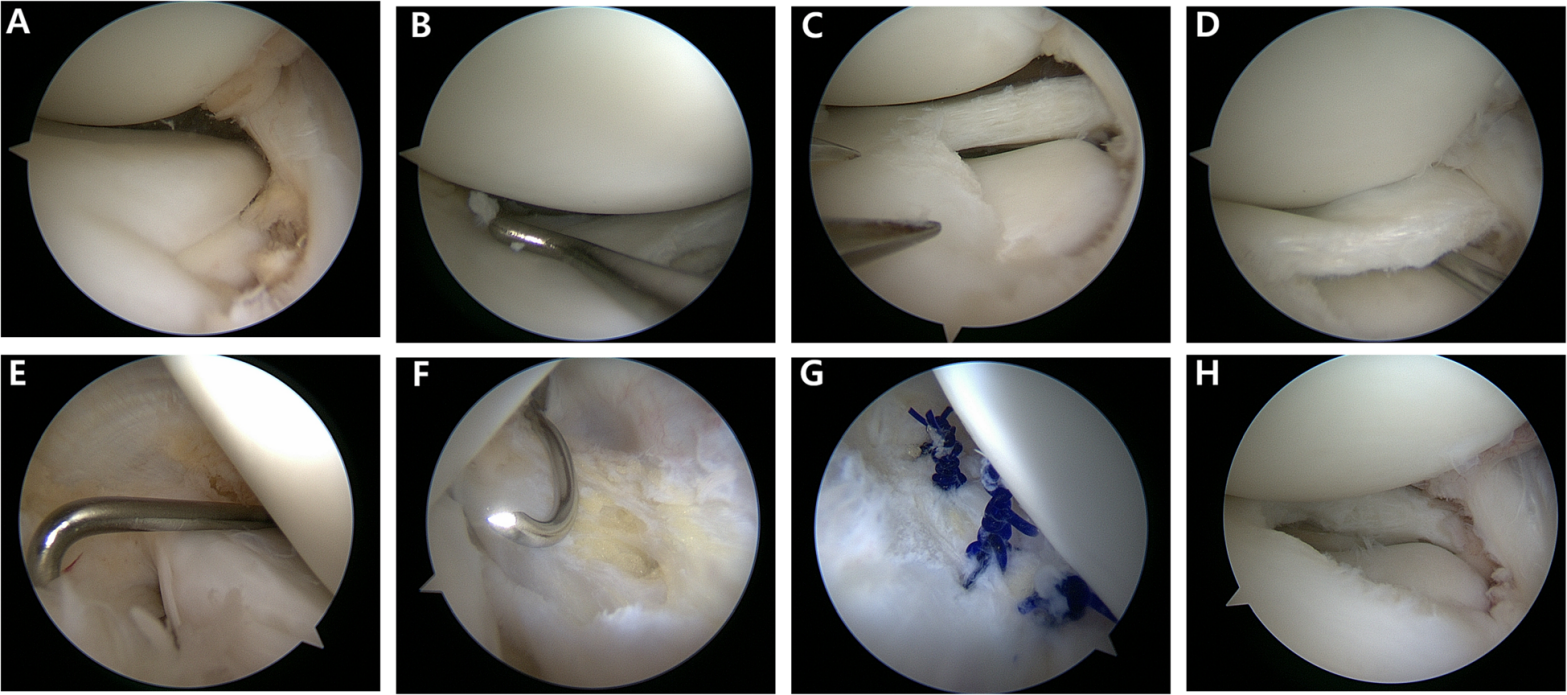
Arthroscopic view recorded during treatment of the anterocentral shift type of complete discoid lateral meniscus (DLM) on the right knee of a 12-year old girl. Reduction of the torn DLM was possible with an arthroscopic probe (**a**, **b**). Partial meniscectomy was performed with the midbody of the medial meniscus as the reference point (**c**). As the longitudinal tear in the posterior horn leads to instability (**d**, **e**), meniscal repair using a modified all-inside technique with a suture hook was performed to achieve stabilization (**e**–**h**)

Meniscal repair is necessary in patients with unstable DLM owing to peripheral detachment during arthroscopy; however, the repair is surgically demanding especially when surgeons unexpectedly face peripheral detachment. Thus, surgical planning and preparation are essential when treating patients with DLM; the classification proposed by Ahn et al. [5] provides useful information to surgeons for predicting the peripheral detachment of the DLM on MRI. Our preferred technical pearls, which are based on the classification proposed by Ahn et al., are as follows [4]: Our preferred principle for DLM repair is (1) reduction first, (2) reshaping DLM with reference to the midbody of the medial meniscus, and finally, (3) repair. Occasionally, meniscal morphology is distorted due to meniscal shifting; thus, 1-stitch sutures are used for reduction in cases with posterocentral or central shifting before partial meniscectomy. When the posterolateral corner loss of the DLM is considerable and reduction with a probe is impossible, subtotal meniscectomy is reluctantly considered. Once the partial meniscectomy is completed, the suture repair of the peripheral tear is performed. A posterolateral portal is used in cases requiring posterior horn examination or repair. All repairs are performed using No. 0 polydioxanone (PDS) absorbable sutures (Ethicon, Somerville, NJ) with a stitch interval of approximately 3–4 mm. Suture techniques are determined based on the location and extent of peripheral tear considering accessibility and handiness. To repair tears in the anterior horn to the midbody, a modified outside-in suture technique is performed using a suture hook (Linvatec, Largo, FL) with a spinal needle preloaded with No. 0 Maxon (Covidien, Mansfield, MA, USA) to pull out the sutures (Fig. 4) [9]. To repair tears in the midbody to the posterolateral corner, a modified inside-out suture technique is performed using a zone-specific cannula and double-arm needles [8]. To repair tears in the posterolateral corner to the posterior horn, a modified all-inside suture technique is performed using a suture hook via a posterolateral portal (Fig. 5) [6]. Meniscal repair even in the most posterior zone and popliteal zone can achieved with the modified all-inside suture technique using the posterolateral portal. Postoperatively, crutches were used for non-weight bearing for 4 weeks and partial weight bearing for additional 4 weeks. Also, range of motion gradually increased 2-days after surgery with a brace and reached 120° of knee flexion by 8 weeks. Running was permitted 3 months after surgery, and kneeling was avoided for 6 months. At 5–6 months after surgery, return to sports was allowed when recoveries of strength and neuromuscular coordination was confirmed, although we cautiously recommend not to perform high-impact sports activity considering the high risk of re-tear and avoidance of tear in the asymptomatic contralateral knee with DLM.

Meniscal allograft transplantation may be considered a treatment option in symptomatic patient after subtotal or total meniscectomy of DLM and some authors have described good clinical results [36, 72]; however, long-term studies are need to establish the durability of the results.

#### Clinical outcomes

Studies have reported favorable clinical outcomes after both partial and subtotal meniscectomy in patients with DLM during the short-, mid-, and long-term follow-up; however, degenerative changes have been noted in subtotal meniscectomy [2, 4, 10, 11, 34, 39, 47, 55, 56, 71]. Thus, several systematic reviews on DLM treatment have been reported recently [46, 48, 63]. Lee et al. [48] performed a systematic review of the long-term surgical outcomes of DLM in 2017, including 11 studies involving 422 DLM cases. The pooled studies in the systematic review included arthroscopic partial meniscectomy with/without meniscal repair, subtotal meniscectomy, and total meniscectomy with a minimum of 5.5 years of follow-up. Good clinical results were shown in most of the included studies, with mild joint space narrowing in the lateral compartment without moderate or severe changes [48]. In 2017, Smuin et al. [63] performed a systematic review of partial versus total meniscectomy of symptomatic DLM during the short- and long-term follow-up. Postoperative clinical outcomes were analyzed by use of the Ikeuchi grading system [29] and classified as excellent (full range of motion, no knee snapping, and no pain), good (infrequent pain with exertion and full range of motion), fair (slight pain, knee snapping on motion, and full range of motion), or poor (constant pain and/or recurrent locking of knee). The four studies included for quantitative synthesis of the short-term outcomes (follow-up < 4 years) showed that 221 of 293 knees (75.3%) demonstrated excellent Ikeuchi outcomes in the partial meniscectomy group, similar to those in the total meniscectomy group (49 of 65 knees, 75.4%) [63]. However, five studies included to assess long-term outcomes (follow-up ≥4 years) showed that 277 of 517 knees (53.6%) in the partial meniscectomy group demonstrated excellent Ikeuchi outcomes, which differed significantly from total meniscectomy group (70 of 183 knees, 38.2%) (*P* < .001) [63]. Lee et al. [46] performed a systematic review and meta-analysis of the clinical and radiographic results of partial versus total meniscectomy in patients with symptomatic DLM. They included eight studies and found significantly higher proportions of cases with normal cartilage status or mild chondral wear (grade 0 or 1 of the Tapper and Hoover classification) for partial meniscectomy than for total meniscectomy (228 of 261 [87.4%] vs. 94 of 169 [55.6%], odds ratio [OR] 9.08; *P* < .001) [46]. However, the clinical outcomes were similar between the two groups [46]. Based on the clinical and radiographic results of recent systematic reviews, partial meniscectomy should be the first treatment option in patients with symptomatic DLM.

#### Prognostic factors

A recent systematic review reported that older age at surgery, longer follow-up period, and subtotal or total meniscectomy were risk factors for degenerative change after surgical treatment of the DLM [48]. Another recent review article on the DLM reported that younger patients were more likely to achieve satisfactory clinical results and that, compared to partial meniscectomy, subtotal or total meniscectomy led to increased contact pressure on the cartilage, resulting in degenerative changes [35]. Additionally, correlation and logistic regression analysis have identified the following prognostic factors: (1) re-tear is associated with younger age and open growth plate [59]; (2) higher Lysholm knee score is related to shorter duration of symptoms prior to surgery and larger varus alignment [45] as well as age < 10 years [71]; (3) degenerative changes in the lateral compartment are associated with older age [16, 59], high body mass index (BMI, ≥30 kg/m^2^) [59], subtotal meniscectomy (vs. partial meniscectomy with or without repair) [2], and other meniscal tear types (vs. horizontal tear) [16]; (4) preoperative meniscal shifting is a risk factor for decreased remaining meniscal width, representing a potential risk for degenerative changes (Table 1).

**Table 1:** Prognostic factors for the treatment outcomes of the DLM identified using correlation or multivariate logistic regression analysis

Author (year)	No. of knees	Mean F/U (years)	Dependent variables	Independent variables	Odds ratio (95% CI)	*P* value
Ahn et al. (2015) [2]	48	10.1	Degenerative changes	Subtotal meniscectomy (vs. partial meniscectomy)	13.56 (1.26–145.92)	0.028
Cho et al. (2019) [16]	355	–	ICRS grades 2–4 of LFC	Horizontal tear	0.02 (0–0.16)	0.009
				Age (per year increase)	1.04 (1.03–1.06)	< 0.001
			ICRS grades 2–4 of LTP	Horizontal tear	0.26 (0.13–0.49)	< 0.001
				Age (per year increase)	1.03 (1.02–1.05)	< 0.001
Kim et al. (2019a, b) [37]	48	1.1	Reduction of remaining meniscal width	Preoperative meniscal shift	12.0 (1.59–90.74)	0.016
Lee et al. (2018) [45]	73	10	Clinical outcomes (Lysholm)	Duration of symptoms prior to surgery	−0.129^a^	0.003
				Last follow-up FTA	1.362^a^	0.045
Sabbag et al. (2019) [59]	59	> 2	Re-tear	Age (per year increase)	0.96 (0.93–0.99)^b^	0.01
				Open growth plates (vs closed)	3.19 (1.15–8.88)^b^	0.03
			Degenerative changes	Age (per year increase)	1.02 (1.00–1.05)	0.046
				BMI ≥30 kg/m^2^ (vs. < 30)	3.75 (1.43–9.84)	< 0.01
Yoo et al. (2015) [71]	48	10.1	Clinical outcomes (Lysholm)	Age < 10 years (vs, ≥10)	2.37 (1.1–5.1)	0.032

*BMI* body mass index, *CI* confidence interval, *DLM* discoid lateral meniscus, *FTA* femorotibial angle, *F/U* follow-up, *ICRS* International Cartilage Repair Society, *LFC* lateral femoral condyle, *LTP* lateral tibial plateau

^a^Unstandardized coefficients of ß

^b^Hazard ratio

## Conclusion

The DLM is an anatomical variation that knee surgeons frequently encounter. The prevalence of the DLM is higher among the Asians than among other races, and both knees are often involved. Although patients with the DLM are often asymptomatic, the DLM is frequently associated with meniscal tears and related symptoms, such as pain, snapping, and limited extension, because the DLM is larger and thicker and its ultrastructural quality is poor; hence, is prone to tearing compared to the normal menisci. Given these characteristics, surgeons should carefully examine and perform close follow-up of not only the affected knee but also the contralateral side (considering the bilateral involvement) of patients presenting no or subtle symptoms. Simple radiographs may provide indirect signs of DLM; however, MRI is necessary for confirming DLM and for surgical decision-making with respect to symptomatic patients. The MRI classification proposed by Ahn et al. may provide useful information for surgical planning as the classification is based on the peripheral detachment of the DLM. Partial meniscectomy with or without meniscal repair has shown favorable clinical outcomes and protects against degenerative changes compared to subtotal or total meniscectomy during the short- and long-term follow-up. Thus, preserving the meniscus as much as possible using various meniscal suture techniques should be considered in the treatment of patients with DLM. Our preferred principle for DLM treatment is reduction first, followed by reshaping DLM with reference to the midbody of the medial meniscus and as firm repair as possible.

## References

[1] Adachi N, Ochi M, Uchio Y, Kuriwaka M, Shinomiya R (2004). Torn discoid lateral meniscus treated using partial central meniscectomy and suture of the peripheral tear. Arthroscopy.

[2] Ahn JH, Kim KI, Wang JH, Jeon JW, Cho YC, Lee SH (2015). Long-term results of arthroscopic reshaping for symptomatic discoid lateral meniscus in children. Arthroscopy.

[3] Ahn JH, Lee SH, Yoo JC, Lee HJ, Lee JS (2010). Bilateral discoid lateral meniscus in knees: evaluation of the contralateral knee in patients with symptomatic discoid lateral meniscus. Arthroscopy.

[4] Ahn JH, Lee SH, Yoo JC, Lee YS, Ha HC (2008). Arthroscopic partial meniscectomy with repair of the peripheral tear for symptomatic discoid lateral meniscus in children: results of minimum 2 years of follow-up. Arthroscopy.

[5] Ahn JH, Lee YS, Ha HC, Shim JS, Lim KS (2009). A novel magnetic resonance imaging classification of discoid lateral meniscus based on peripheral attachment. Am J Sports Med.

[6] Ahn JH, Oh I (2006). Arthroscopic all-inside lateral meniscus suture using posterolateral portal. Arthroscopy.

[7] Ahn JH, Wang JH, Kim DU, Lee DK, Kim JH (2017). Does high location and thickness of the Wrisberg ligament affect discoid lateral meniscus tear type based on peripheral detachment?. Knee.

[8] Ahn JH, Wang JH, Oh I (2004). Modified inside-out technique for meniscal repair. Arthroscopy.

[9] Ahn JH, Wang JH, Yoo JC, Kim SK, Park JH, Park JW (2006). The modified outside-in suture: vertical repair of the anterior horn of the meniscus after decompression of a large meniscal cyst. Knee Surg Sports Traumatol Arthrosc.

[10] Ahn JY, Kim TH, Jung BS, Ha SH, Lee BS, Chung JW, Kim JM, Bin SI (2012). Clinical results and prognostic factors of arthroscopic surgeries for discoid lateral menisci tear: analysis of 179 cases with minimum 2 years follow-up. Knee Surg Relat Res.

[11] Aichroth PM, Patel DV, Marx CL (1991). Congenital discoid lateral meniscus in children. A follow-up study and evolution of management. J Bone Joint Surg (Br).

[12] Andrish JT (1996). Meniscal injuries in children and adolescents: diagnosis and management. J Am Acad Orthop Surg.

[13] Araki Y, Ashikaga R, Fujii K, Ishida O, Hamada M, Ueda J, Tsukaguchi I (1998). MR imaging of meniscal tears with discoid lateral meniscus. Eur J Radiol.

[14] Atay OA, Pekmezci M, Doral MN, Sargon MF, Ayvaz M, Johnson DL (2007). Discoid meniscus: an ultrastructural study with transmission electron microscopy. Am J Sports Med.

[15] Bisicchia S, Tudisco C (2013). Re-growth of an incomplete discoid lateral meniscus after arthroscopic partial resection in an 11 year-old boy: a case report. BMC Musculoskelet Disord.

[16] Cho WJ, Kim JM, Lee BS, Kim HJ, Bin SI (2019). Discoid lateral meniscus: a simple horizontal tear was associated with less articular cartilage degeneration compared to other types of tear. Knee Surg Sports Traumatol Arthrosc.

[17] Clark CR, Ogden JA (1983). Development of the menisci of the human knee joint. Morphological changes and their potential role in childhood meniscal injury. J Bone Joint Surg Am.

[18] Cui JH, Min BH (2007). Collagenous fibril texture of the discoid lateral meniscus. Arthroscopy.

[19] Dashefsky JH (1971). Discoid lateral meniscus in three members of a family. Case reports. J Bone Joint Surg Am.

[20] Dickason JM, Del Pizzo W, Blazina ME, Fox JM, Friedman MJ, Snyder SJ (1982). A series of ten discoid medial menisci. Clin Orthop Relat Res.

[21] Fairbank TJ (1948). Knee joint changes after meniscectomy. J Bone Joint Surg (Br).

[22] Fleissner PR, Eilert RE (1999). Discoid lateral meniscus. Am J Knee Surg.

[23] Fukuta S, Masaki K, Korai F (2002). Prevalence of abnormal findings in magnetic resonance images of asymptomatic knees. J Orthop Sci.

[24] Gebhardt MC, Rosenthal RK (1979). Bilateral lateral discoid meniscus in identical twins. J Bone Joint Surg Am.

[25] Greis PE, Bardana DD, Holmstrom MC, Burks RT (2002). Meniscal injury: I. Basic science and evaluation. J Am Acad Orthop Surg.

[26] Ha CW, Jang JW, Kim M, Na SE, Lee HJ, Park YB (2017). The utility of the radiographic condylar cut-off sign in children and adolescents with complete discoid lateral meniscus. Knee Surg Sports Traumatol Arthrosc.

[27] Ha CW, Lee YS, Park JC (2009). The condylar cutoff sign: quantifying lateral femoral condylar hypoplasia in a complete discoid meniscus. Clin Orthop Relat Res.

[28] Han SB, Babu CP, Choi JH, Suh DW, Jang KM (2018). Regeneration of lateral discoid meniscus after arthroscopic partial meniscectomy in an adult patient. Knee Surg Sports Traumatol Arthrosc.

[29] Ikeuchi H (1982). Arthroscopic treatment of the discoid lateral meniscus. Technique and long-term results. Clin Orthop Relat Res.

[30] Jeannopoulos CL (1950). Observations on discoid menisci. J Bone Joint Surg Am.

[31] Jeon SW, Choi CH, Jung M, Chun YM, Kim SJ, Jin S, Kim SH (2019). The fate of the contralateral knee in patients with a lateral discoid meniscus. Arthroscopy.

[32] Jordan MR (1996). Lateral meniscal variants: evaluation and treatment. J Am Acad Orthop Surg.

[33] Kaplan EB (1957). Discoid lateral meniscus of the knee joint; nature, mechanism, and operative treatment. J Bone Joint Surg Am.

[34] Kelly BT, Green DW (2002). Discoid lateral meniscus in children. Curr Opin Pediatr.

[35] Kim JG, Han SW, Lee DH (2016). Diagnosis and treatment of discoid meniscus. Knee Surg Relat Res.

[36] Kim JM, Bin SI (2006). Meniscal allograft transplantation after total meniscectomy of torn discoid lateral meniscus. Arthroscopy.

[37] Kim SH, Ahn J, Kim TW, Kim KI, Lee SH (2019). Midbody of the medial meniscus as a reference of preservation in partial meniscectomy for complete discoid lateral meniscus. Knee Surg Sports Traumatol Arthrosc.

[38] Kim SJ, Lee YT, Kim DW (1998). Intraarticular anatomic variants associated with discoid meniscus in Koreans. Clin Orthop Relat Res.

[39] Kinugasa K, Hamada M, Yonetani Y, Matsuo T, Mae T, Nakata K, Horibe S (2019). Discoid lateral meniscal repair without saucerization for adolescents with peripheral longitudinal tear. Knee.

[40] Klingele KE, Kocher MS, Hresko MT, Gerbino P, Micheli LJ (2004). Discoid lateral meniscus: prevalence of peripheral rim instability. J Pediatr Orthop.

[41] Kocher MS, DiCanzio J, Zurakowski D, Micheli LJ (2001). Diagnostic performance of clinical examination and selective magnetic resonance imaging in the evaluation of intraarticular knee disorders in children and adolescents. Am J Sports Med.

[42] Kocher MS, Logan CA, Kramer DE (2017). Discoid lateral meniscus in children: diagnosis, management, and outcomes. J Am Acad Orthop Surg.

[43] Kramer DE, Micheli LJ (2009). Meniscal tears and discoid meniscus in children: diagnosis and treatment. J Am Acad Orthop Surg.

[44] Kushare I, Klingele K, Samora W (2015). Discoid meniscus: diagnosis and management. Orthop Clin North Am.

[45] Lee CR, Bin SI, Kim JM, Lee BS, Kim NK (2018). Arthroscopic partial meniscectomy in young patients with symptomatic discoid lateral meniscus: an average 10-year follow-up study. Arch Orthop Trauma Surg.

[46] Lee DH, D’Lima DD, Lee SH (2019). Clinical and radiographic results of partial versus total meniscectomy in patients with symptomatic discoid lateral meniscus: a systematic review and meta-analysis. Orthop Traumatol Surg Res.

[47] Lee DH, Kim TH, Kim JM, Bin SI (2009). Results of subtotal/total or partial meniscectomy for discoid lateral meniscus in children. Arthroscopy.

[48] Lee YS, Teo SH, Ahn JH, Lee OS, Lee SH, Lee JH (2017). Systematic review of the long-term surgical outcomes of discoid lateral meniscus. Arthroscopy.

[49] Moser MW, Dugas J, Hartzell J, Thornton DD (2007). A hypermobile Wrisberg variant lateral discoid meniscus seen on MRI. Clin Orthop Relat Res.

[50] Nathan PA, Cole SC (1969). Discoid meniscus. A clinical and pathologic study. Clin Orthop Relat Res.

[51] Papadopoulos A, Kirkos JM, Kapetanos GA (2009). Histomorphologic study of discoid meniscus. Arthroscopy.

[52] Park YB, Ha CW, Jang JW, Kim M, Lee HJ, Park YG (2018). Prediction models to improve the diagnostic value of plain radiographs in children with complete discoid lateral meniscus. Arthroscopy.

[53] Patel NM, Cody SR, Ganley TJ (2012). Symptomatic bilateral discoid menisci in children: a comparison with unilaterally symptomatic patients. J Pediatr Orthop.

[54] Pellacci F, Montanari G, Prosperi P, Galli G, Celli V (1992). Lateral discoid meniscus: treatment and results. Arthroscopy.

[55] Raber DA, Friederich NF, Hefti F (1998). Discoid lateral meniscus in children. Long-term follow-up after total meniscectomy. J Bone Joint Surg Am.

[56] Rao PS, Rao SK, Paul R (2001). Clinical, radiologic, and arthroscopic assessment of discoid lateral meniscus. Arthroscopy.

[57] Rao SK, Sripathi Rao P (2007). Clinical, radiologic and arthroscopic assessment and treatment of bilateral discoid lateral meniscus. Knee Surg Sports Traumatol Arthrosc.

[58] Restrepo R, Weisberg MD, Pevsner R, Swirsky S, Lee EY (2019). Discoid meniscus in the pediatric population:: emphasis on MR imaging signs of instability. Magn Reson Imaging Clin N Am.

[59] Sabbag OD, Hevesi M, Sanders TL, Camp CL, Dahm DL, Levy BA, Stuart MJ, Krych AJ (2019). High rate of recurrent meniscal tear and lateral compartment osteoarthritis in patients treated for symptomatic lateral discoid meniscus: a population-based study. Orthop J Sports Med.

[60] Samoto N, Kozuma M, Tokuhisa T, Kobayashi K (2002). Diagnosis of discoid lateral meniscus of the knee on MR imaging. Magn Reson Imaging.

[61] Silverman JM, Mink JH, Deutsch AL (1989). Discoid menisci of the knee: MR imaging appearance. Radiology.

[62] Smillie IS (1948). The congenital discoid meniscus. J Bone Joint Surg (Br).

[63] Smuin DM, Swenson RD, Dhawan A (2017). Saucerization versus complete resection of a symptomatic discoid lateral meniscus at short- and long-term follow-up: a systematic review. Arthroscopy.

[64] Soejima T, Kanazawa T, Tabuchi K, Noguchi K, Inoue T, Murakami H (2013). Regeneration of ring-shaped lateral meniscus after partial resection of discoid meniscus with anterior cruciate ligament reconstruction. Int J Surg Case Rep.

[65] Stanitski CL (1998). Correlation of arthroscopic and clinical examinations with magnetic resonance imaging findings of injured knees in children and adolescents. Am J Sports Med.

[66] Stein MI, Gaskins RB, Nalley CC, Nofsinger C (2013). Regeneration of a discoid meniscus after arthroscopic saucerization. Am J Orthop.

[67] Vandermeer RD, Cunningham FK (1989). Arthroscopic treatment of the discoid lateral meniscus: results of long-term follow-up. Arthroscopy.

[68] Watanabe MTS, Ikeuchi (1979). Atlas of arthroscopy.

[69] Yamasaki S, Hashimoto Y, Takigami J, Terai S, Takahashi S, Nakamura H (2017). Risk factors associated with knee joint degeneration after arthroscopic reshaping for juvenile discoid lateral meniscus. Am J Sports Med.

[70] Yaniv M, Blumberg N (2007). The discoid meniscus. J Child Orthop.

[71] Yoo WJ, Jang WY, Park MS, Chung CY, Cheon JE, Cho TJ, Choi IH (2015). Arthroscopic treatment for symptomatic discoid meniscus in children: midterm outcomes and prognostic factors. Arthroscopy.

[72] Yoon KH, Lee SH, Park SY, Jung GY, Chung KY (2014). Meniscus allograft transplantation for discoid lateral meniscus: clinical comparison between discoid lateral meniscus and nondiscoid lateral meniscus. Arthroscopy.

[73] Young RB (1889). The external semilunar cartilage as a complete disc. Memoirs and memoranda in anatomy.

